# A Persistent Solid Pseudopapillary Tumor of the Pancreas: Case Report and Brief Literature Review

**DOI:** 10.1089/crpc.2015.29006.jbz

**Published:** 2015-11-01

**Authors:** Jin Bao Zhang, Dong Shang, Theresa P. Yeo, Shawnna Cannaday, Warren R. Maley, Charles J. Yeo

**Affiliations:** ^1^ Department of Surgery, The First Affiliated Hospital of Dalian Medical University, Dalian, China.; ^2^ Department of Surgery, Thomas Jefferson University Hospital, Philadelphia, Pennsylvania.

**Keywords:** Hamoudi tumor, pancreas, pancreatic tumors, solid pseudopapillary neoplasm, solid pseudopapillary tumor

## Abstract

**Background:** Solid pseudopapillary tumors (SPTs) of the pancreas are uncommon neoplasms, first reported in 1934, well described by Frantz in 1959, and later further characterized by Hamoudi in 1970. Ninety percent of these tumors occur in young females in their second to third decade of life. An interesting case of a persistent solid pseudopapillary neoplasm is described in this report.

**Case presentation:** A 24-year-old woman from a Middle Eastern country presented with an 8.2 × 7.6 cm heterogeneous-enhancing lesion growing within the uncinate process of the pancreas. She had first experienced symptoms at the age of 12 years. Imaging studies showed that the mass closely abutted the superior mesenteric vein as well as the superior mesenteric artery (SMA). The patient underwent an open cholecystectomy and a classic pancreaticoduodenectomy. During the resection, the SMA was transected due to tumor adherence. The vessel was subsequently reapproximated in an end-to-end manner. On the first postoperative day, thrombosis of the SMA occurred and a bile leak developed. The patient returned to the operating room for SMA embolectomy and for repair of a hepaticojejunostomy leak, with redo of the biliary-enteric anastomosis. Histopathological examination showed solid pseudopapillary-arranged cells and cystic areas, showing strong cellular immunoreactivity for CD56, CD10, vimentin, and β-catenin, and weak diffuse staining for synaptophysin. The tumor stained negative for chromogranin A, trypsin, AE1/AE3, and E-cadherin. Molecular genetic analysis was negative for the *MYB* gene deletion. At nearly 1 year of follow-up, the patient is well with no evidence of tumor recurrence.

**Conclusion:** SPTs of the pancreas should be included in the differential diagnosis of pancreatic tumors, especially in young women.

## Introduction and Background

Solid pseudopapillary tumors (SPTs) of the pancreas are a rare neoplasm accounting for 0.13–2.76% of all pancreatic tumors.^1^ An SPT was first reported in 1933,^2^ was well described by Frantz in 1959,^3^ later characterized by Hamoudi in 1970,^4^ and finally accepted as an entity by the World Health Organization in 1996.^5^ The tumor is often described by its eponyms: “Frantz” tumor or “Hamoudi” tumor. Ninety percent of SPTs occur in young women in their second to third decade of life.^6^ One hypothesis posits that the tumor is related to sex hormones, although this theory has not been confirmed. Surgical resection is the mainstay of therapy and an adequate surgical resection generally results in long-term tumor-free survival. SPTs can be quite different from other pancreatic tumors in radiological and pathological findings. The cellular origin of SPTs and tumorigenesis, while now thought to be embryonic in origin, remains an enigma.

## Case Presentation

A 24-year-old woman from a Middle Eastern country presented to the Jefferson Pancreas, Biliary and Related Cancer Center for evaluation of a recurrent pancreatic mass. She complained of right upper quadrant fullness, and physical examination revealed a remote right subcostal incision. At the age of 12 years, she had first developed decreased appetite, weight loss, fatigue, pruritus, and subsequently became jaundiced. Medical records from that episode revealed that an endoscopic biliary stent was placed with surgical exploration through a right subcostal incision and partial resection/enucleation of a pancreatic mass. In the intervening 12 years, the mass had persisted and enlarged, although the patient was asymptomatic, having neither anorexia, pruritus, nor jaundice.

**FIG. 1.**
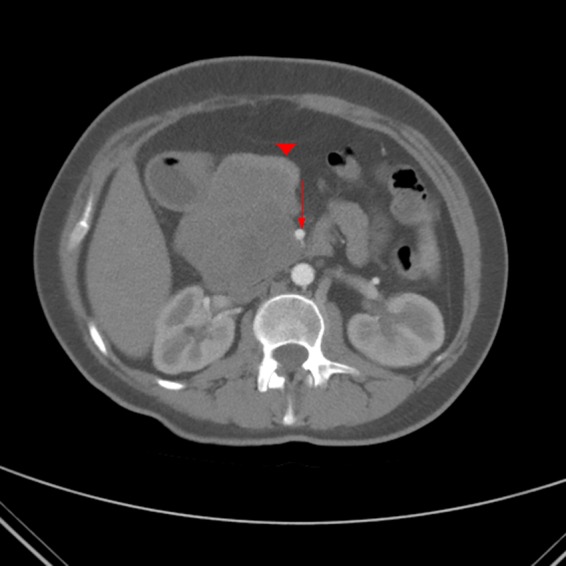
Preoperative contrast-enhanced abdominal computed tomography (CT) scan, axial arterial phase. The images reveal a low-density mass (*red arrow head*) measuring about 8.2 × 7.6 cm in diameter and prominently involving the uncinate process of the pancreas. The pancreatic head and neck are displaced and splayed around the anterior aspect of the tumor, certainly abutting the superior mesenteric artery (SMA; *red arrow*).

Routine hematology and basic chemistry panels were normal. The tumor marker cancer antigen 19-9 was mildly elevated at 89 U/mL (<35 U/mL). An abdominal computed tomography (CT) scan with contrast revealed an 8.2 × 7.6 cm heterogeneous-enhancing lesion, prominently involving the uncinate process of the pancreas (Fig. 1). The pancreatic head and neck were displaced and splayed around the anterior aspect of the tumor. The mass abutted the superior mesenteric vein (SMV) as well as the superior mesenteric artery (SMA). There was no evidence of main pancreatic ductal dilatation and the pancreatic neck, body, and tail were normal. Imaging showed no evidence of metastatic disease to the liver or regional lymph nodes. The mass was believed to be an SPT, based on the previous partial resection and the accompanying pathology report.

The patient underwent an open cholecystectomy and a difficult classic pancreaticoduodenectomy. The operative time was 12 h and the estimated intraoperative blood loss was 1500 mL. There was no evidence of metastasis, but the tumor had adhered extensively to the SMV and portal vein and surrounded the SMA. We were able to accomplish the separation of the tumor from the venous structures without incident; however, separating the tumor from the SMA proved challenging. At one point, the SMA was transected due to adherence of the tumor. The SMA was subsequently reapproximated in an end-to-end manner with good arterial Doppler signals distal to the anastomosis.

**FIG. 2.**
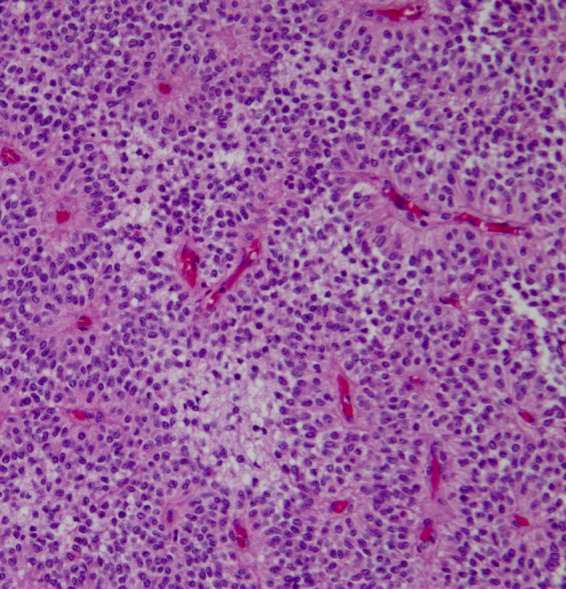
Histopathological photomicrographs of a solid pseudopapillary tumor (SPT). The image shows that the small round cells arranged and formed into nests, pseudopapillae, and microcysts. Note the tumor cells are traversed by a delicate vascular network. (Hematoxylin and eosin, original magnification, ×20).

Pathological analysis of the surgical specimen revealed the tumor to be a solid pseudopapillary neoplasm (Fig. 2). All surgical margins were free of neoplasia and all harvested regional lymph nodes showed only follicular lymphoid hyperplasia, with no evidence of granulomas or neoplasia. Immunohistochemical stains of the specimen were positive for CD56, CD10, and vimentin, with the neoplastic cells showing strong diffuse nuclear and cytoplasmic staining for β-catenin and weak diffuse staining for synaptophysin. The neoplastic cells were negative for chromogranin A, trypsin, AE1/AE3, and E-cadherin. Molecular genetic analysis was negative for the *MYB* gene deletion.

**FIG. 3.**
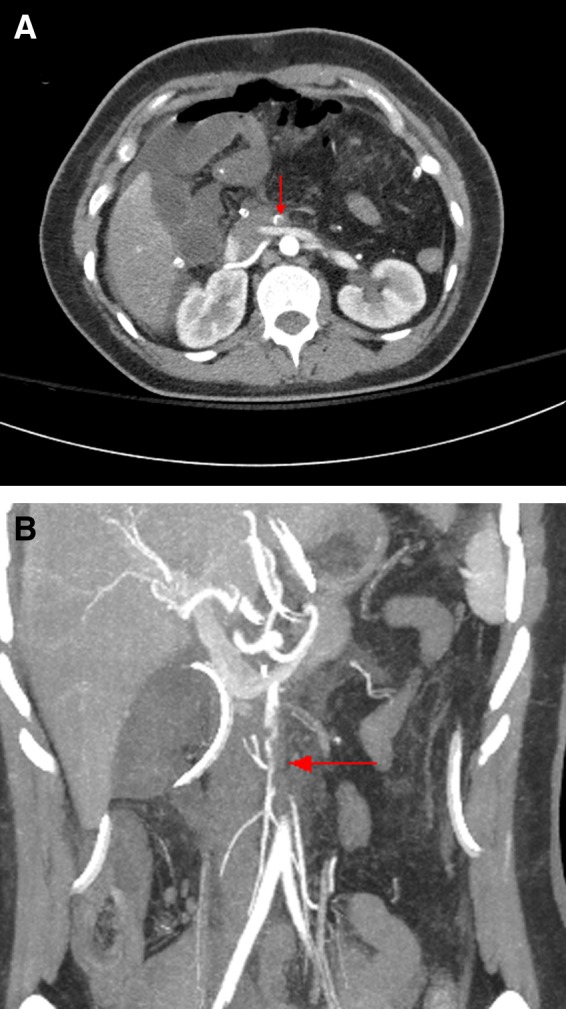
**(A, B)** Contrast-enhanced abdominal CT scan on postoperative day 1. A = axial. B = coronal. The images reveal an intraluminal thrombus (*red arrow*) in the proximal SMA ∼1.5 cm from its aortic origin, causing near complete occlusion of the SMA.

On the first postoperative day, the patient had a small amount of bile visible in the operatively placed drains, she was fluid seeking, and her abdomen was somewhat distended. Due to the suspicion of a vascular insult related to the SMA reconstruction, an abdominal CT with intravenous contrast was obtained and revealed an intraluminal thrombus in the proximal SMA, ∼1.5 cm from its origin off the aorta, causing near complete occlusion of the SMA (Fig. 3). She was therefore returned to the operating room where the proximal jejunum appeared ischemic. We performed an SMA embolectomy and repaired a leak at her hepaticojejunostomy through reconstruction of the biliary-enteric anastomosis. She tolerated the reoperation well and improved nicely.

On the fourth postoperative day, an upper gastrointestinal series with water-soluble contrast instilled into the stomach through a nasogastric tube revealed no contrast extravasation, and both the afferent and efferent limbs of the duodenojejunostomy were grossly patent.

The patient and her family were instructed on the home management of the large abdominal incision and superficial wound infection. Healing occurred over the next 4 months. Telehealth monitoring was used by our nursing experts to communicate with the patient on a regular basis, with mobile phone images documenting the status of the wound.

She returned to Philadelphia for a follow-up visit after 6 months. At that time, the patient appeared well, her wound was completely healed, and an abdominal CT scan with contrast showed normal after pancreaticoduodenectomy anatomy, without any evidence of recurrent or persistent tumor.

## Discussion

SPTs are most often located at the head of the pancreas,^7^ as in the case herein reported. SPTs have a low-grade malignant potential and a good prognosis.^8^ Interestingly, SPTs can mimic malignant pancreatic tumors with hypermetabolism of FDG on positron emission tomography (PET) scanning.^9^ Typically, the radiographical appearance of SPTs is a well-defined encapsulated tumor that contains a mixture of solid and cystic components. In fact, most patients are asymptomatic with no abnormalities or physical signs, and the lesion is generally detected incidentally on imaging or physical examination. The size of the mass and its location (compressing adjacent organs) may lead to vague abdominal discomfort. Obstruction of the bile duct or intestine, jaundice, pancreatitis, or spontaneous tumor rupture are rare occurrences. Nakamura et al.^10^ described a rare case of SPT, which resulted in occlusion of the splenic vein, leading to the development of gastric varices and left-sided extrahepatic portal hypertension.

Surgical resection of SPTs is the mainstay of therapy, and a complete surgical extirpation most often results in long-term survival. Almost 95% of SPTs can be cured by complete excision with margin-negative resection.^11^ Interestingly, Nakahara et al. described an unusual case of SPT in a young woman refusing surgery.^12^ During a 10-year follow-up period, the maximum diameter of the tumor gradually decreased from 4.5 to 1.5 cm, and the authors posited that some degenerative changes such as hemorrhage and necrosis, followed by absorption, resulted in the natural shrinkage of the tumor. Goh et al.^13^ suggest that the malignant potential of SPTs is difficult to predict by gender, age, or tumor size without surgery or biopsy, but that the diagnosis of SPTs should be suspected when a young female has a mass of pancreatic origin. In addition, if one finds a large, centrally calcified, well-encapsulated, mixed cystic/solid mass by CT/MRI, the diagnosis of an SPT should be suspected.

On hematoxylin–eosin staining, SPTs are histologically characterized by solid pseudopapillary-arranged cells and cystic areas. SPTs may often mimic endocrine tumors of the pancreas, with morphological features including nest formation and tumor cells with eccentric nuclei. The characteristic immunohistochemical features of SPTs are CD10, CD56, and vimentin positivity. Importantly, beta-catenin has nuclear expression in all reported cases of SPTs.^14^ In general, the neoplastic cells in SPTs are negative for chromogranin A. Occasionally, as in our case, immunoreactivity for synaptophysin is seen. In our case, the immunohistochemical findings were positive for CD56, CD10, vimentin, and β-catenin, and weakly positive for synaptophysin. However, the tumor was negative for chromogranin A and trypsin and also negative for AE1/AE3 and E-cadherin. In addition, the molecular genetics showed the tumor to be negative for the MYB gene deletion (the MYB gene family plays an essential role in cell growth, differentiation, and apoptosis). Chromogranin A and synaptophysin are both general markers for neuroendocrine tumors and are typically reported together. In our case, chromogranin A was negative, and there was only weak diffuse staining for synaptophysin, indicating that the tumor contained scattered neuroendocrine cells. CD56 is also a marker for neuroendocrine lesions, but synaptophysin is more specific and less sensitive to neuroendocrine differentiation. Burford et al.^15^ suggest that fine-needle aspiration samples tested for expression of CD10 and the characteristic expression of E-cadherin/β-catenin are highly specific for distinguishing SPTs from pancreatic endocrine neoplasms. Cytokeratins (AE1/AE3) are intermediate filament (IF) proteins used for the cytoskeleton. AE1/AE3 are expressed and regulated in most epithelial cells. These markers are expressed in many tumors and may be helpful in identifying the origin of a neoplasm. In certain situations, IF vimentin is a useful marker to assess the primary site of a tumor and it has a relationship with dedifferentiation and carcinogenesis in the epithelial cells, suggesting that it may be a useful marker for pancreatic SPT precursor cells.

## Conclusion

In conclusion, SPTs have characteristic pathological and radiological features and should be included in the differential diagnosis of pancreatic tumors, especially in young women.

## Abbreviations Used

18F-FDG: β-2-[18F]-Fluoro-2-deoxy-d-glucose
AE1/AE3: cytokeratins
CT: computed tomography
IF: intermediate filament
MRI: magnetic resonance imaging
PET: positron emission tomography
SMA: superior mesenteric artery
SMV: superior mesenteric vein
SPT: solid pseudopapillary tumor
